# Association of Perfluoroalkyl and polyfluoroalkyl substances (PFASs) exposures and the risk of systemic lupus erythematosus: a case–control study in China

**DOI:** 10.1186/s12940-023-01019-1

**Published:** 2023-11-07

**Authors:** Yan He, Can Qu, Jing Tian, Justyna Miszczyk, Hua Guan, Ruixue Huang

**Affiliations:** 1 Department of Ophthalmology, The Second Xiangya Hospital, Central South University, Changsha, 410011 Hunan China; 2 Hunan Clinical Research Center of Ophthalmic Disease, Changsha, 410011 Hunan China; 3 Department of Occupational and Environmental Health, Xiangya School of Public Health, Central South University, Changsha, 410078 Hunan Province China; 4 Department of Rheumatology and Immunology, The Second Xiangya Hospital, Central South University, Changsha, 410011 Hunan China; 5 Department of Experimental Physics of Complex Systems, The H. Niewodniczański Institute of Nuclear Physics Polish Academy of Sciences, Kraków, Poland; 6 Department of Radiation Biology, Beijing Key Laboratory for Radiobiology,, Beijing Institute of Radiation Medicine, Beijing, 100850 China

**Keywords:** PFASs, SLE, Association, Risk

## Abstract

Perfluoroalkyl and polyfluoroalkyl substances (PFASs) may have a role in impaired health. However, the data on the association between PFASs and Systemic lupus erythematosus (SLE) have been limited. We designed a population-based case–control study in China and evaluated the association. 100 normal persons (Control) and 100 SLE patients (Case) were obtained from 113 controls and 125 cases according to matching conditions. Serum samples were collected by venipuncture for UHPLC-MRM-MS Analysis to obtain the concentration of five PFASs in participants. Demographic characterization description was performed for the two groups of participants, the PFASs concentration distribution of the two groups was described and compared, then divided into three tiers (< 50th, 50th ~ 75th, > 75th) for subsequent analysis. Conditional logistic regression models were utilized to calculate the odds ratios (ORs) and 95% CIs for SLE. Relationship between changes in the concentration of PFASs and the risk of SLE assessed by restricted cubic spline. As the highest serum levels of the five PFASs tested in this study population, the highest perfluoroundecanoic acid (PFUnA) quartile had a 2.78-fold (95%CI: 1.270, 6.10) compared with the lowest quartile of PFUnA exposure, other types of PFASs also showed high association with SLE as well as PFASs mixture. Additionally, the exposure of PFASs exist a dose–response relationship (ptrend < 0.05). This risk association remained be found after adjusting the covariates in model 1 (adjustment of BMI) and in model 2(adjustment of BMI, smoking, drinking, hypertension and leukocyte). The restricted cubic spline illustrated a gradual increase in the possible risk of SLE with the increasing exposure of PFASs components levels. Our study firstly revealed that PFASs are risk factors for SLE and PFASs exposures are associated with SLE risk in a dose − response manner. Evidence from larger and more adequately powered cohort studies is needed to confirm our results.

**Supplementary Information:**

The online version contains supplementary material available at 10.1186/s12940-023-01019-1.

## Introduction

Systemic lupus erythematosus (SLE), characterized with immune system auto-reactivity, autoantibody production and immune complex deposition, is a life-threatening chronic disorder [1]. The prevalence of SLE was approximately 73 to 178 persons per 100,000 observed in the USA [2]. Furthermore, the annual mean per-person medical cost for SLE was about US$17,000 and prescription costs were US$5000, which both of costs significantly higher than non-SLE respondents [2]. As of lacking effective therapy methods to reverse and control the SLE injury, it is critical important to figure out potential risk factors for prevention of SLE at the earliest possible.

With the rapid development of population, the requirement for agriculture, industrial and medical products have been tremendously increased across the world. This greatly leads to the larger increasing amount of environmental pollutants, which pose the essential risk for health [3, 4], of which some of pollutants (e.g., air pollution, pesticides, ultraviolet radiation) have been reported serving as major role in occurrence of SLE risk [5]. Currently, a growing attention that environmental pollutants Perfluoroalkyl and polyfluoroalkyl substances (PFASs) may be risk for immune system damage. PFASs are a group of anthropogenic pollutants of global concern, consumption of contaminated food and drinking water is the main source of systemic human intake, followed by inhalation and dermal absorption [6]. Of the more than 9,000 recognized substances [7], only perfluorooctane sulfonic acid (PFOS) and perfluorooctanoic acid (PFOA) typically have regulatory thresholds or guideline values [8]. Other PFASs are classified as "substances of very high concern" by the European Commission [Registration, Evaluation, Authorization and Restriction of Chemicals (REACH)] due to their bioaccumulation, environmental persistence and toxicity [9, 10]. Here we focus on five PFASs, perfluoroundecanoic acid (PFUnA), perfluorododecanoic acid (PDA), heptafluorobutyric acid (PFBA), perfluoroheptanoic acid (PFHpA), and perfluorohexanesulfonic acid (PFHxS). These PFASs were detected at higher rates and concentrations in this survey population. Long-chain PFAS (C > 8) tend to have longer biological half-lives than short-chain PFASs [11]. PFUnA and PDA are long-chain PFASs, and some studies have shown that these long-chain PFASs are more toxic. PFBA is a short-chain PFAS containing four carbons, but it has been shown that in autopsy evaluations of individuals unknowingly exposed to PFASs through environmental and consumer products, higher concentrations of PFBA were detected in the lungs and kidneys compared to long-chain PFASs [12], which raises concerns about the potential toxicity of PFBA [13]. Swedish adolescents have very low concentrations of PFHpA, probably due to the low concentration of PFHpA in food [14], but PFHpA is also strongly associated with human health, with one study showing a statistically significant increase in PFHpA in individuals with coronary heart disease [15]. PFHxS is classified as a short-chain PFASs and can be found in drinking water up to a maximum concentration of 7.1 ng/L [16], but reached 978.5 ng/L in Baiyangdian, as well as 1,487 ng/L in the Fuhe River, China, and the high level of exposure raises concerns about the damage of this PFASs to population health [17]. Exposure of PFASs is associated with overall risk of infectious disease [18], activating inflammation through innate immune system [19], increasing risk of childhood allergy [20] and contributing to risk of type 1 diabetes in offspring [21]. However, limited epidemiological and laboratory cell and animal experiments have directly study the association between PFASs and SLE risk. Meanwhile, evidence have shown PFASs are widely existing in our ecosystems [22]. In terms of the increasing incidence of SLE from unknown causes, wee hypothesized that PFASs may be associated with the risk of SLE. Therefore, in this study, we designed a case–control study in Hunan Province, Central South University to analysis the association between PFASs exposure and SLE risk.

## Methods

### Study design and data collection

This study was designed as a population-based case–control study based on our previous human investigation study performed in Hunan Province, Central China from July 2016 to Sep 2017 [23]. This investigation has indicated that environmental pollutants, heavy metals are associated with increased liver damage, we then continue investigate whether the emerging environmental pollutants Perfluoroalkyl and polyfluoroalkyl substances (PFASs) were associated with SLE. Thus, between March 2019 to May 2022, the Second Xiangya Hospital, Central South University in Changsha, China recruited 125 patients with SLE through the following inclusion criteria, (i) aged over 18 years; (ii) newly diagnosed with SLE either with outpatient claims or upon hospitalization [24]; (iii) these who had other complications including diabetes, acute infection diseases were excluded; (iv) approval for the study and sign their written informed consent prior to the survey. Exclusion criteria included concurrent pregnancy, use of hormonal or other medications in the recent 1 month. We defined SLE group patients based on the Systemic Lupus International Collaborative Clinics (SLICC) classification criteria [25]. We defined controls from the same hospital as healthy population without clinical and laboratory evidence of SLE or other dysfunction, a total of 113 eligible healthy individuals were recruited in this survey. Then the control participants were randomly selected and matched for age (± 3 years), sex taken from the general population (desired case − control ratio 1:1), 25 excluded in the case group and 13 excluded in the control group due to unmatched age and gender appropriateness. Finally, a total of 100 cases and 100 controls were included in this investigation. The study was approved by Medical ethics committee of the Second Xiangya Hospital, Central South University (No. LYF2021028).

Baseline information for participants were recruited using in-person interviews. Through the interview, gender, age, weight, lifestyle (e.g., smoking and alcohol drinking) and chronic medical history such as hypertension were recorded. Body mass index (BMI) was calculated by the formula of weight (kg)/height (m2). The cut-off values of BMI for adults in China are as follows: < 18.5 kg/m2, underweight; 18.5 − 23.9 kg/m2, normal; 24.0 − 27.9 kg/m2, overweight; and ≥ 28.0 kg/m2, obesity. Blood biochemistry values including Leukocyte, Hemoglobin, Platelets and Lymphocyte were detected. The normal Leukocyte value ranges from 3.5 × 109 to 9.5 × 109, normal Platelets value ranges from 125 × 109 to 350 × 109, normal Lymphocyte value ranges from 1.1 × 109 to 3.2 × 109, normal Hemoglobin value ranges from 130 to 175 g/L for man and from 115 to 150 g/L for female.

### Measurements of the target five PFASs

Blood samples were recruited by Department of Ophthalmology, The Second Xiangya Hospital, Central South University. Serum samples were collected through venipuncture with one 10 ml red top Vacutainer tube per participant. Blood samples were centrifuged at 3,000 rpm for 20 min. taking a disposable pipet, the serum was transferred to 1.5 ml polypropylene tubes. Samples were stored at -80 °C until analysis by shipping to Biotree, Ltd. Shanghai, China.

Five PFASs, including PFUnA, PDA, PFBA, PFHpA, and PFHxS, were determined using the ultra-high performance liquid chromatography (UHPLC). They are co-existing in serum, and this analysis of the association of their respective concentrations with SLE. In order to investigate the effect of the mixture of PFASs on SLE, we added the measured concentrations of the five PFASs to obtain the total concentration for further analysis. The UHPLC separation was carried out using an EXIONLC System (Sciex), equipped with a Waters ACQUITY UPLC BEH C18 column (100 × 2.1 mm, 1.7 μm, Waters). The mobile phase A was 3 mmol/L NH4OH and 3 mmol/L ammonium acetate in water, and the mobile phase B was acetonitrile. The column temperature was set at 40 ℃. The auto-sampler temperature was set at 4 ℃ and the injection volume was 5 μL. A SCIEX 6500 QTRAP + triple quadrupole mass spectrometer (Sciex), equipped with an IonDrive Turbo V electrospray ionization (ESI) interface, was applied for assay development. Typical ion source parameters were: Curtain Gas = 30 psi, IonSpray Voltage = -4500 V, temperature = 400 ℃, Ion Source Gas 1 = 45 psi, Ion Source Gas 2 = 45 psi. The MRM parameters for each of the targeted analytes were optimized using flow injection analysis, by injecting the standard solutions of the individual analytes, into the API source of the mass spectrometer. Several most sensitive transitions were used in the MRM scan mode to optimize the collision energy for each Q1/Q3 pair (Table S1). Among the optimized MRM transitions per analyte, the Q1/Q3 pairs that showed the highest sensitivity and selectivity were selected as ‘quantifier’ for quantitative monitoring. The additional transitions acted as ‘qualifier’ for the purpose of verifying the identity of the target analytes. SCIEX Analyst Work Station Software (Version 1.6.3) and Multiquant 3.03 software were employed for MRM data acquisition and processing.

The calibration standard solution was diluted stepwise, with a dilution factor of 2. These standard solutions were subjected to UHPLC-MRM-MS analysis. The signal-to-noise ratios (S/N) were used to determine the lower limits of detection (LLODs) and lower limits of quantitation (LLOQs). The LLODs and LLOQs were defined as the analyte concentrations that led to peaks with signal-to-noise ratios (S/N) of 3 and 10, respectively, according to the US FDA guideline for bioanalytical method validation.

The precision of the quantitation was measured as the relative standard deviation (RSD), determined by injecting analytical replicates of a QC sample. The accuracy of quantitation was measured as the analytical recovery of the QC sample determined. The percent recovery was calculated as [(mean observed concentration) / (spiked concentration)] × 100%. The PFASs and biochemical indices of the blood samples of the cases and controls of this study were measured in the same batch, and the systematic bias was controlled by repeating the measurements three times.

### Statistical analysis

The differences between the control group and SLE group was conducted by t-test, chi-square test or Mann–Whitney U test. Calculate Pearson correlation coefficients and *P* values for two-by-two groups of five PFASs. Conditional logistic regression models were utilized to calculate the odds ratios (ORs) and 95% CIs for SLE. Levels of PFASs were categorized into three groups, with less than the 50th quantile as one group, greater than 50th and less than the 75th quantile as the second group, and greater than the 75th quantile as the final group. The lowest quartile was subjected to the reference group. Two models were used to adjust the covariates based on the known and suspected SLE factors. Model1 was adjusted for BMI, whereas model 2 was adjusted for smoking, drinking, hypertension and leukocyte. Furthermore, the restricted cubic splines were illustrated based on the 5th, 25th, 50th, 75th and 95th percentiles of the levels. In the spline models, we adjusted for model 1. SPSS 25.0 (SPSS, Chicago, IL, USA) software and R (version 4.0.2) were used for statistical analysis in this study.

## Results

### LC Separation and analysis metrics

**Fig. 1:**
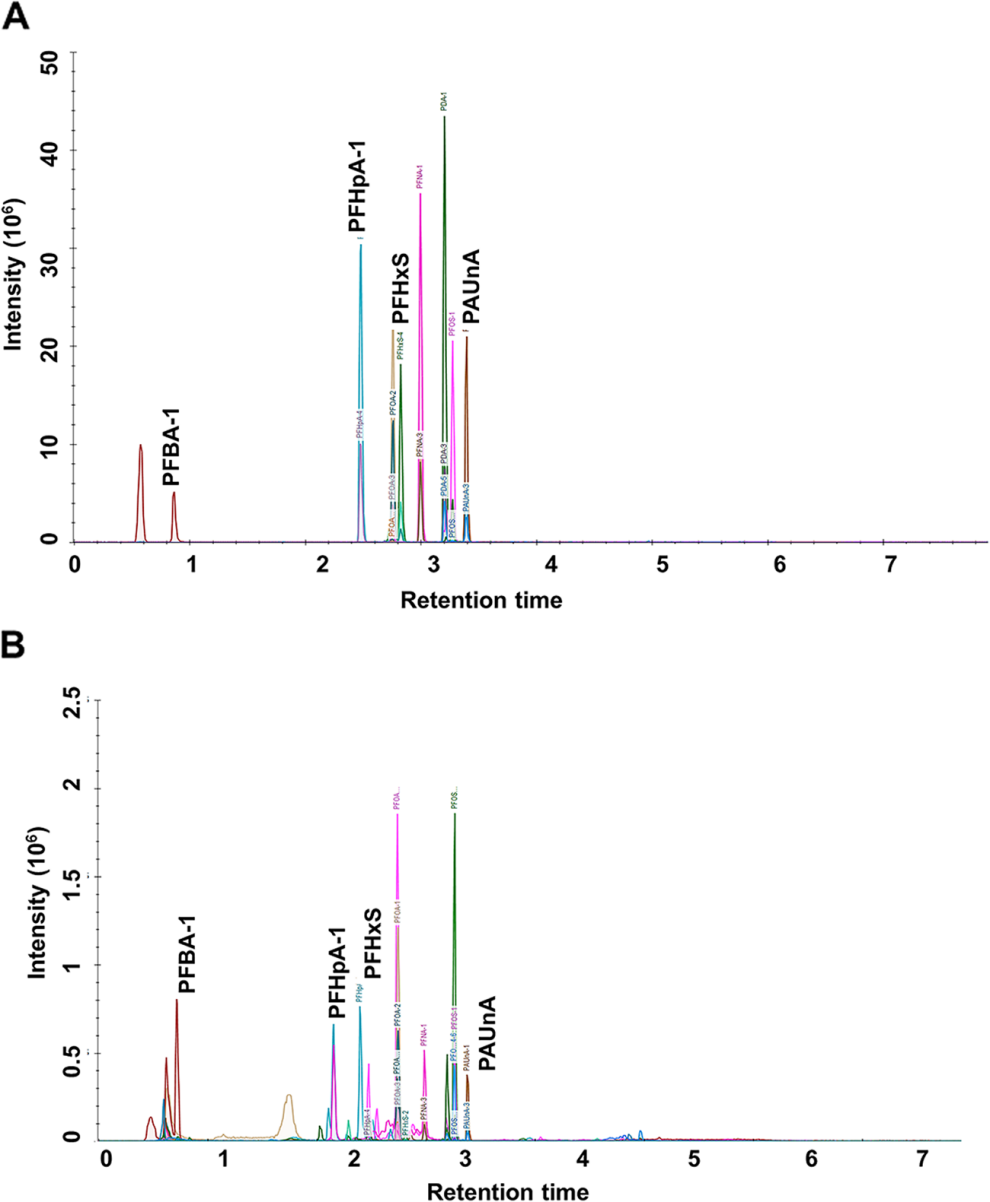
The extracted ion chromatographs (EICs) from a standard solution (Fig. 1**A**) and a sample (Fig. 1**B**)

Figure 1 shows the extracted ion chromatographs (EICs) from a standard solution (Fig. 1A) and a sample (Fig. 1B) of the targeted analytes under the optimal conditions. As can be seen from this figure, (i) all of the analytes showed symmetrical peak shapes, (ii) baseline separations were obtained, (iii) and the retention time and peak shapes for all of the analytes showed good correlation between the standard solution and the real sample. Table S2 lists the resulting lower-limits of detection and quantitation (LLODs and LLOQs), and the LLODs ranged from 0.05 to 3.13 nmol/L, the LLOQs ranged from 0.10 to 6.25 nmol/L for the plant hormones. Correlation coefficients (R2) of regression fitting were above 0.9956 for all the analytes, indicating a good quantitative relationship between the MS responses and the analyte concentrations, which was satisfying for targeted metabolomics analysis. The analysis metrics indicated that the method allowed accurate quantitation of the targeted metabolites in the biological sample, in the concentration range described as above.

### Baseline information of enrolled participants

In total, 200 participants were enrolled into the study (Con group, 100 and SLE group, 100, respectively). The main baseline information was illustrated in Table 1. The average age was 43.93 and 44.08 years for control and SLE groups, respectively. The female-to-male ratio was approximately 5:1 in each group. A difference was found for Leukocyte value within the two groups (*p* = 0.005). Nevertheless, no significance was found with regards to the Body mass index (BMI), hypertension, smoking, drinking .

**Table 1:** Demographic characteristics of study participants and comparison of systemic lupus erythematosus cases and the control group odds ratios (OR) with 95% confidence intervals (CIs)

	control group(*n* = 100)	case group(*n* = 100)	p^a^
Age	43.93 ± 9.52	44.08 ± 9.97	0.91^b^
Gender			
male	16(16.0%)	16(16.0%)	1.00
female	84(84.0%)	84(84.0%)	
Body mass index			0.47
< 18.0	0(0.0%)	2(2.0%)	
18.0–23.9	71(71.0%)	74(74.0%)	
24.0–27.9	21(21.0%)	17(17.0%)	
≥ 28.0	8(8.0%)	7(7.0%)	
Hypertension			0.82
yes	10(10.0%)	11(11.0%)	
no	90(90.0%)	89(89.0%)	
Smoking			0.19
yes	15(15.0%)	9(9.0%)	
no	85(85.0%)	91(91.0%)	
Drinking			
yes	22(22.0%)	19(19.0%)	0.60
no	78(78.0%)	81(81.0%)	
Leukocyte			
abnormal	19(19.0%)	37(37.0%)	0.005
normal	81(81.0%)	63(63.0%)	
Hemoglobin			
abnormal	33(33.0%)	65(65.0%)	< 0.001
normal	67(67.0%)	35(35.0%)	
Platelets			0.005
abnormal	6(6.0%)	19(19.0%)	
normal	94(94.0%)	81(81.0%)	
Lymphocyte			0.026
abnormal	27(27.0%)	42(42.0%)	
normal	73(73.0%)	58(58.0%)	

^a^From Mann − Whitney U test

^b^From student-t test

### Perfluoroalkyl and polyfluoroalkyl substances (PFASs) Exposure

Table 2 presents the serum PFAS level distribution and detection rate. For five components, the detection rates were 96.5% for Perfluoroundecanoic acid (PFUnA), 99.2% for Perfluorodecanoic acid (PDA), 100% for Heptafluorobutyric acid (PFBA), 95.9% for Perfluoroheptanoic acid (PFHpA), 98.6% for Perfluorohexane sulfonic acid (PFHxS), respectively. SLE patients had higher concentrations of this five serum PFASs components than control normal participants as shown in Table 2. For example, PFUnA concentration was higher in SLE (0.88 nmol/L) than in control normal group (0.70 nmol/L) (*p* = 0.014). PDA concentration was higher in SLE (0.46 nmol/L) than in control normal group (0.38 nmol/L) (*p* < 0.001). We added the five PFASs together as this PFASs mixture to carry out the correlation analysis with SLE, PFASs concentration was higher in SLE (2.05 nmol/L) than in control normal group (1.56 nmol/L) (*p* < 0.001). These results indicated that exposure of PFASs may be associated with the SLE.

**Table 2:** Distribution and Detection Rate of PFAS

			Percentile						
exposure	group	mean	5th	25th	50th	75th	95th	Detection rate (%)	p^a^
PFUnA	total	0.79	0.05	0.23	0.82	1.22	1.54	96.5%	0.014
	case	0.88	0.09	0.54	0.99	1.27	1.54		
	control	0.70	0.05	0.17	0.66	1.15	1.54		
PDA	total	0.42	0.05	0.21	0.38	0.55	1.07	99.2%	0.018
	case	0.46	0.06	0.27	0.39	0.63	1.07		
	control	0.38	0.05	0.15	0.34	0.51	1.05		
PFBA	total	0.27	0.06	0.14	0.23	0.34	0.65	100%	< 0.001
	case	0.31	0.04	0.16	0.27	0.41	0.74		
	control	0.22	0.06	0.13	0.21	0.28	0.43		
PFHpA	total	0.17	0.03	0.08	0.13	0.22	0.54	95.9%	< 0.001
	case	0.21	0.05	0.11	0.16	0.23	0.60		
	control	0.14	0.01	0.06	0.11	0.18	0.34		
PFHxS	total	0.15	0.03	0.07	0.12	0.19	0.36	98.6%	< 0.001
	case	0.19	0.05	0.11	0.17	0.23	0.52		
	control	0.11	0.02	0.05	0.09	0.13	0.30		
PFASs mixture	total	1.80	0.58	1.24	1.82	2.32	2.98		< 0.001
	case	2.05	0.70	1.66	2.08	2.55	3.05		
	control	1.56	0.50	1.10	1.49	2.06	2.63		

^a^From Mann − Whitney U test

We analyzed and calculated the correlation coefficients and P values for each of the five PFAS of serum. The results showed that in the total population (Table S3), PFUnA correlated with PDA with a correlation coefficient and *P* value of 0.15 (0.03) and PFHpA correlated with PFHxS for a correlation value of 0.14 (0.04). The correlation of PFUnA with PDA remained only in the case group with a correlation value of 0.25 (0.01) was shown in the Table S4, and that of PFHpA with PFHxS was retained only in the controls with a correlation value of 0.21 (0.04) was shown in the Table S5. The remaining PFAS correlations were not statistically significant.

### Association between PFASs exposure and SLE

**Fig. 2:**
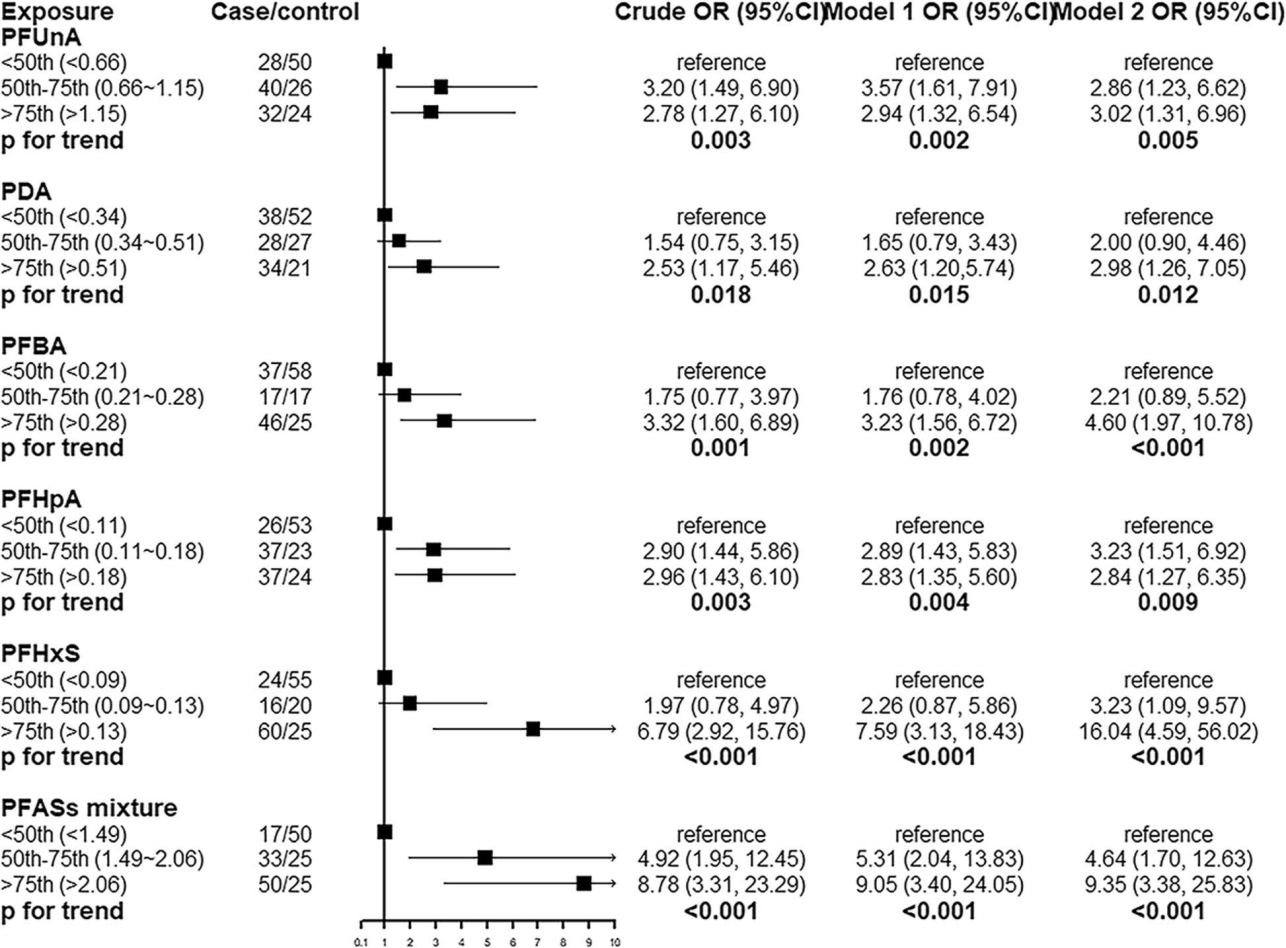
Odds ratios (ORs) with 95% confidence intervals (CIs) for the risk of SLE by quartiles of PFASs concentrations based on the levels of the control group. Conditional logistic regression analysis with the exposure divided into three groups: 0-50%, 50-75%,and 75-100%. Model 1 was adjusted for BMI and model 2 was adjusted for model 1, smoking, drinking, hypertension and leukocyte

**Fig. 3:**
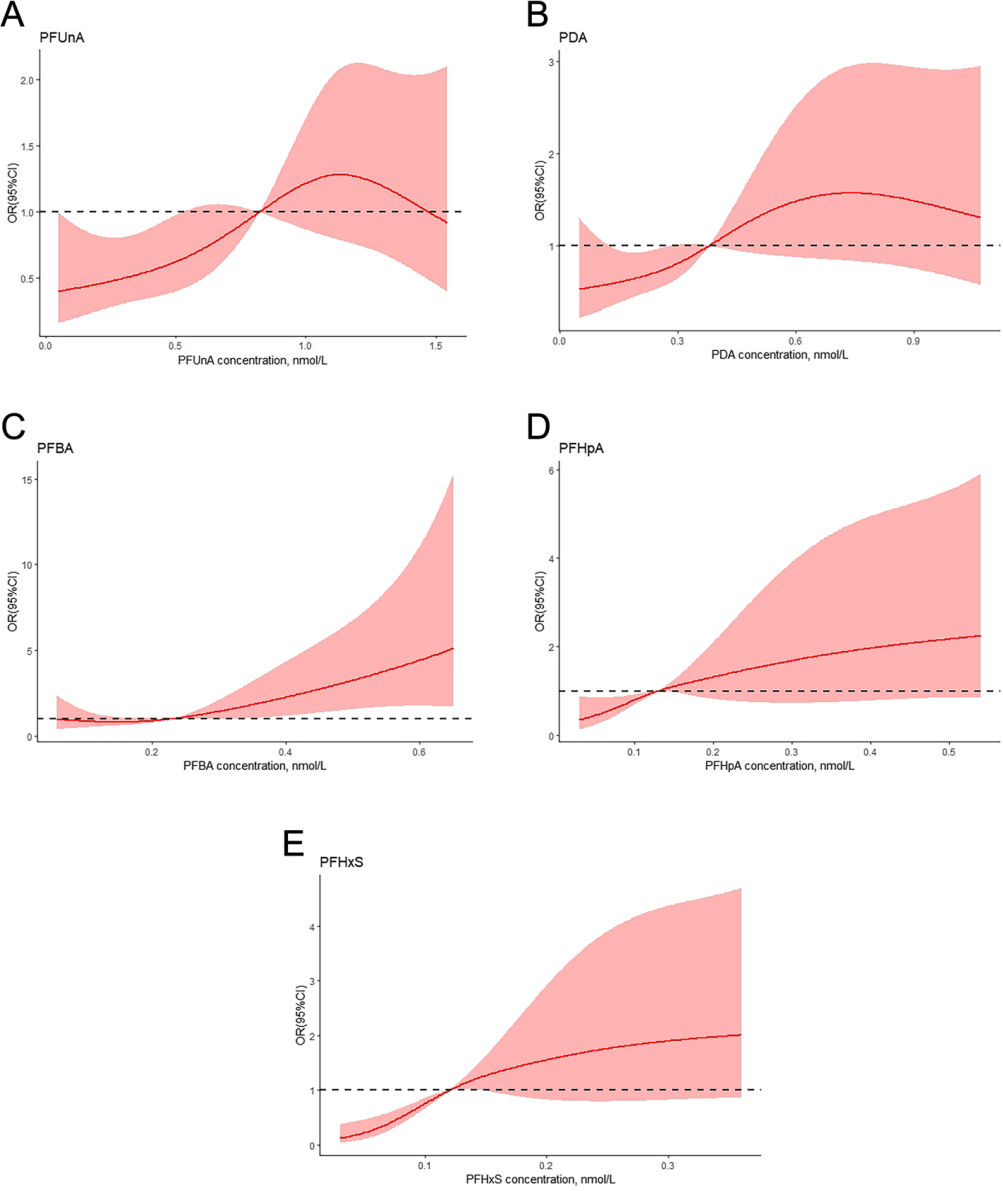
Restricted cubic spline plots: (**A**) The association between PFUnA and SLE risk; (**B**) the association between PDA and SLE risk; (**C**). The association between PFBA and SLE risk; (**D**) The association between PFHpA and SLE risk; (**E**) The association between PFHxS and SLE risk. The solid line with color represents the odds ratio (OR) for SLE. The colored part represents 95% confidence intervals. Odds ratios (ORs) for SLE calculated according to continuous PFASs values, adjusted by Model 1. The knots were placed at the 5th, 25th, 50th, 75th, and 95th percentiles for each PFASs distribution

Figure 2 presents the effects of PAFSs components on SLE. The possible risk of SLE in the highest PFUnA quartile was 2.78 times (95%CI: 1.27, 6.10) higher than the lowest quartile. The possible risk of SLE in the highest PDA quartile was 2.53 times (95%CI: 1.17, 5.46) higher than the lowest quartile The possible risk of SLE in the highest PFBA quartile was 3.32 times (95%CI: 1.60, 6.89) higher than the lowest quartile. The possible risk of SLE in the highest PFHpA quartile was 2.96 times (95%CI:1.43, 6.10) The possible risk of SLE in the highest PFHxS quartile was 6.79 times (95%CI: 2.92, 15.76) higher than the lowest quartile. The percentile 50th to 75th percentile of PFUnA and PFHpA had a greater risk of being associated with SLE compared to those less than the 50th percentile, with OR (95% CI) of 3.20 (1.49, 6.90) and 2.90 (1.44, 5.86), respectively. There was no statistically significant difference in the percentile 50th to 75th quartiles of the remaining three PFSAs compared to the risk of SLE in the less than 50th quartile. PFASs mixture and SLE also demonstrated a significant correlation in this analysis. The risk association remained be found after adjusting the covariates in model 1 (adjustment of BMI) and in model 2(adjustment of BMI, smoking, drinking, hypertension and leukocyte). After adjustment for model 2, ORs for SLE were almost always increased and equally statistically significant. The P for trend for the five PFASs under various model adjustments as well as the unadjusted case after trend test were statistically significant, indicating a trend in the changes between quartiles. Forest plots are shown with unadjusted crude OR (95% CI). The restricted cubic spline illustrated a gradual increase in the possible risk of SLE with the increasing exposure of PFASs components levels (Fig. 3). In particular, PFBA had a statistically significant increase in OR with SLE with increasing concentration. The other four PFASs also had a trend of increasing ORs with gradually increasing concentrations but were not statistically significant.

## Discussion

To the best of our knowledge, our report is the first to analysis the association between PFASs exposure and SLE risk. Our study indicated that five PFASs components were risk factors for SLE with a dose–response manner. Our findings provide further evidence of the positive effects of environmental pollution on SLE risk.

Typically, people expose PFASs through multiple resources including food packing material, fir fighting foams, paper and textile coatings, drinking water and air or house dust widely in our daily life [26]. Hiroaki et al. had reported that the median serum PFHxS concentration was 0.94 ng/mL [27], but the report from Watkins et al. was almost 3 times higher of 2.6 ng/ml [28]. Elyse et al. did not detect the PFBA in human serum in their study but Dan et al. found that PFBA concentration was 1.45 ng/mL [28]. PFHpA serum level was reported as 0.056 ng/mL by Qian et al. [29] but was higher of 1.412 ng/mL in Liao Q et al. study [30]. Our study has indicated that the average content of tested compounds in normal population serum ranks in the order PFUnA (0.70 nmol/L) > PDA (0.38 nmol/L) > PFBA (0.22 nmol/L) > PFHpA (0.14 nmol/L > PFHxS (0.11 nmol/L), respectively. The differences between our results and previous studies may be due to the different population, different detection methods or the sample size. Nevertheless, our study offers evidence that the serum concentration of PFASs increased although at low levels and may potential for the SLE risk. Taken together, we speculated that the primary mode of PFASs exposure may the environment. However, the detail environmental routine should be further evaluated the concentration ratio of air, water, soil or foods.

There is no direct epidemiological evidence to report the association between PFASs and SLE risk. However, there are reports on the association of PFASs of other diseases’ risks. For example, it has been found that PFASs were associated with impaired kidney function [31], risk of gestational diabetes mellitus [32] and hypertension [33]. It has also reported that exposure of PFUnA and PDA were associated with risk of diabetes [34] and children’s adiposity [35] and ocular conditions [36]. It has been found exposure of PFHpA was associated with decreased couple fecundity [37] and risk of cardiovascular diseases [38]. Exposure of PFHxS may be associated with increased susceptibility to liver injury in children [39] and risk for endometriosis and uterine leimyoma [40] and risk of autism spectrum disorder [41]. Our study has indicated that the OR values of tested compounds in the order PFHxS (OR, 6.79, 95%CI, 2.92–15.76) > PFBA (OR, 3.32, 95%CI, 1.60–6.89) > PFHpA (OR, 2.96 95%CI, 1.43–6.10) > PFUnA (OR, 2.78, 95%CI, 1.27–6.10) > PDAA (OR, 2.53, 95%CI, 1.17–5.46), respectively. Our study is consistent with these of study that found a positive association between PFASs exposure and human diseases’ risk.

Human SLE is an autoimmune disease characterized by the production of autoantibodies against nuclear and cytoplasmic antigens, accompanied by complement activation, dramatic longitudinal fluctuations in serum C4 and C3 levels, and immune-mediated tissue damage [42]. The aetiology and pathogenesis of SLE is complex and involves a wide range of genetic and environmental factors affecting the onset and progression of the disease and the response to therapy [43]. Biological mechanisms linking PFASs exposure to immune diseases such as SLE remain unclear. The mechanism studies between PFASs and immune diseases are more focused on perfluorooctanesulfonic acid (PFOS), perfluorooctanoate (PFOA), etc. or the whole PFASs mixture., while other PFASs related studies are less, we can speculate the possible biological mechanism of SLE with the five PFASs in this study from similar substances. PFOA and PFOS have been reported to alter adaptive and innate immune responses, including inflammation and cytokine production, in several animal models [44]. It was found that exposure to 100 μM PFOA increased the secretion of cytokines such as tumor necrosis factor-α (TNF-α) and three types of interleukins (IL-1, IL-6, and IL-12), which are closely associated with immune responses [45]. This may be due to the significant increase in glutathione content in macrophages after 100 μM PFOA exposure, allowing macrophages to regulate immune and inflammatory responses by secreting TNF-α, IL-1, IL-6, and IL-12 [46]. A study also demonstrated a significant positive correlation between IgG levels and serum PFOA concentrations [47]. PFASs interact with immune cells and enhance adaptive immune responses of type 2 helper T lymphocytes (Th2) and type 17 helper T lymphocytes (Th17) [48]. Dysregulation of Th17 cellular immunity induces a variety of immunoinflammatory diseases such as rheumatoid arthritis, SLE, psoriasis, systemic sclerosis, and inflammatory bowel disease [49]. More specific processes within the immune system (e.g., calcium signaling) as well as other mechanisms throughout the organism (e.g., lipid metabolism, oxidative stress) may be involved in the immunotoxicity of PFASs [50].

However, some limitations may affect our results explanation. First, the population was from the Second Xiangya Hospital of Central South University in Hunan Province, China, so our findings may have limited generalizability to populations with different exposure characteristics. And only 100 people enrolled into the study, the case–control study instead of cohort study design would also affect the results. Second, baseline information was collected through self-assessment which may generate information bias to affect the results. Third, not all the PFASs were detected in this study which may underestimate the effects of PFASs on SLE. Fourth, we did not systematically assess the sources and routes of PFAS exposure, but only measured serum PFASs concentrations in the study population. A better understanding of the sources of exposure to PFASs would facilitate the development of recommendations that provide scientific initiatives from a public health perspective. Fifth, in addition to PFASs environmental organic pollutants include endocrine disrupting chemicals, phthalates, and many other environmental variables related to human health that were not adequately considered in this study. Despite adjusting for common confounders such as gender and age through pairwise adjustment, and also adjusting for BMI, smoking, alcohol consumption, and leukocytes through modeling, there are still many factors that have not been explored for the risk of SLE and need to be further explored in subsequent investigations. Thus, in the future, we will consider larger and multicenter prospective cohort studies including investigation of sources of exposure and other environmental variables and expanding the sample size to further identified the association of PFASs with SLE risk.

## Conclusions

Our study found that PFASs are risk factors for SLE and PFASs exposures are associated with SLE risk in a dose − response manner. Evidence from larger and more adequately powered cohort studies is needed to confirm our results. Our study may provide a basis for early warning of SLE based on PFASs exposure to assess the risk of SLE.

### Supplementary Information

**Additional file 1.**

## Data Availability

N/A.
