# Supplementary material for: Association of Perfluoroalkyl and polyfluoroalkyl substances (PFASs) exposures and the risk of systemic lupus erythematosus: a case–control study in China
Source: Environ Health. 2023 Nov 7;22:78. doi: 10.1186/s12940-023-01019-1 (PMC10629165; doi:10.1186/s12940-023-01019-1)
Supplement: Supplementary file 1 — Additional file 1. [file 12940_2023_1019_MOESM1_ESM.docx]

| **Abbr.** |  | **Prec Ion** | **Prod Ion** | **Polarity** |
| --- | --- | --- | --- | --- |
| PFUnA | perfluoroundecanoic acid | 212.8 | 168.9 | Negative |
| PDA | Perfluorodecanoic acid | 363.0 | 319.1 | Negative |
| PFBA | Heptafluorobutyric acid | 413.0 | 368.9 | Negative |
| PFHpA | Perfluoroheptanoic acid | 462.8 | 419.0 | Negative |
| PFHxS | Perfluorohexane sulfonate | 398.9 | 99.0 | Negative |

1. **Table S1**. Illustration of specific parameters
2. **Table S2**. Illustration of the resulting lower-limits of detection and quantitation

| **Abbr.** | **LLOD（nmol/L）** | **LLOQ（nmol/L）** | **ULOQ（nmol/L）** | **R^2** |
| --- | --- | --- | --- | --- |
| PFUnA | 0.39 | 0.78 | 200.00 | 0.9996 |
| PDA | 0.10 | 0.20 | 200.00 | 0.9956 |
| PFBA | 0.10 | 0.20 | 12.50 | 0.9994 |
| PFHpA | 0.10 | 0.20 | 200.00 | 0.9988 |
| PFHxS | 0.05 | 0.10 | 200.00 | 0.9996 |

1. **Table S3**. Correlation coefficients and *P* values for each of the five PFAS in the total population.

|  | PFUnA | PDA | PFBA | PFHpA | PFHxS |
| --- | --- | --- | --- | --- | --- |
| PFUnA | 1.00 |  |  |  |  |
| PDA | 0.15 (0.03) | 1.00 |  |  |  |
| PFBA | 0.00 (0.96) | 0.04 (0.53) | 1.00 |  |  |
| PFHpA | 0.02 (0.77) | -0.09 (0.21) | 0.01 (0.94) | 1.00 |  |
| PFHxS | -0.04 (0.54) | 0.06 (0.37) | 0.08 (0.26) | 0.14 (0.04) | 1.00 |

1. **Table S4**. Correlation coefficients and *P* values for each of the five PFAS in the patient group.

|  | PFUnA | PDA | PFBA | PFHpA | PFHxS |
| --- | --- | --- | --- | --- | --- |
| PFUnA | 1.00 |  |  |  |  |
| PDA | 0.25 (0.01) | 1.00 |  |  |  |
| PFBA | -0.04 (0.69) | -0.07 (0.51) | 1.00 |  |  |
| PFHpA | 0.12 (0.89) | -0.13 (0.20) | -0.01 (0.96) | 1.00 |  |
| PFHxS | -0.16 (0.10) | -0.08 (0.43) | 0.01 (0.89) | 0.02 (0.83) | 1.00 |

1. **Table S5**. Correlation coefficients and *P* values for each of the five PFAS in the control group.

|  | PFUnA | PDA | PFBA | PFHpA | PFHxS |
| --- | --- | --- | --- | --- | --- |
| PFUnA | 1.00 |  |  |  |  |
| PDA | 0.02 (0.85) | 1.00 |  |  |  |
| PFBA | -0.05 (0.62) | 0.13 (0.20) | 1.00 |  |  |
| PFHpA | -0.06 (0.58) | -0.11 (0.27) | -0.14 (0.16) | 1.00 |  |
| PFHxS | -0.02 (0.81) | -0.06 (0.58) | 0.20 (0.84) | 0.21 (0.04) | 1.00 |
